# Valedictory Address

**Published:** 1899-08

**Authors:** Clyde S. Hess

**Affiliations:** M’killip Veterinary College


### VALEDICTORY ADDRESS.

DELIVERED BY CLYDE S. HESS,
M'KILLIP VETERINARY COLLEGE, 1899.

EVERY individual of holy ambition ever and anon asks himself the question, "What of my future?" What is true of individuals is equally true of institutions; for institutions are but the organization of human thought. It is, therefore, appropriate on this occasion to ask ourselves the question, What of the future of our college? What are the elements of her strength? Is she worthy of a place side by side of her sister colleges?

Let us recount a few characteristics prophetic of her prosperity. She has many features attractive to persons interested in veterinary science. Prominent among these features is the location. It is situated in the heart of the "Queen of the Northwest," and more accessible to all parts of the country than any other metropolis. It was true in ancient times that "all roads led to Rome." It is true in modern times that all roads lead to Chicago.

Chicago is the nerve-centre of this Republic. Politically, commercially, educationally she is bound (if not already) to occupy the foremost place in this nation. This fact must not be overlooked in a prospective view of our college. It is a product of this fair city, and must, therefore, reflect the spirit of her who gave her birth.

The course of study as conducted in our college is characterized by comprehensiveness, progression and practicability. These characteristics *insure* the perpetuity of an institution.

The spirit of our college is broad and comprehensive, taking into account the needs of the veterinary student. It aims to give him such a survey of veterinary science as to enable him to cope with all the difficulties incident to his profession. It gives him a broad and sure foundation on which to rear the superstructure. It breathes into him a spirit of self-reliance; a spirit of research ; a spirit of independent investigation, making possible his future development, and giving him power to meet the ever-recurring emergencies; or, to express it in familiar terms, it "helps him to help himself."

Says Emerson : "Insist on yourself. Never imitate. Your own gift you can present every moment with the cumulative force of a whole life's cultivation. But if the adopted talent of another you have only an extemporaneous half-possession." Or, as Carlyle puts it : "Be no longer a chaos, but a world or a worldkin. *Produce! produce!* Were it but the pitifullest infinitesimal fraction of a product, in God's name, produce!"

Again, the spirit of our college is progressive. Already she has exemplified this by her expansion of and divergence from the ordinary course of study as offered by sister colleges. For example: Last year there were introduced frequent visits of inspection to the Union Stock-yards. This has proved a source of valuable information. This year a chair was established in the interest of meat- and milk-inspection with a view of preparing students for Government inspectors. And not the least important is the introduction of the morning clinic, which, in itself, is a prophesy of things that are yet to be. It is proposed that a short post-graduate course be offered next year, to consist of lectures, clinics, surgery, and other features of special interest to the practitioner.

Moreover, our college is *practical*, and this is already seen in her present achievements. Her fundamental idea is to bring things to pass—to achieve results. She is not devoted to speculative tendencies, nor to the organization of fine-spun theories; but, on the contrary, she insists on the inculcation of the spirit of practicability ; that is to say, we learn the art of administering medicines by administering them ; we learn the variations of the pulse at the horse's jaw ; we learn the art of dentistry at the horse's head, and learn to distinguish râles and bruits while auscultating the lungs.

In view of the foregoing, we are justified in the assumption that the future of our college will be, like the noonday sun, resplendent with glory.

And now we have met here for the last time. And as the hour draws near for our departure there arises in our hearts a feeling of tender regard for our instructors, who have endeared themselves to us during our college days. Their patience, forbearance, and solicitude for us merit our loving appreçiation.

May I then, for these and other favors, express, in behalf of the class, our gratitude to the instructors and honored president.

Fellow-members of the "Class of '99," this hour finds us in retrospect and prospect. We are lost in memories of the past. We are encircled by hallowed associations. But the past and future are inseparable. Henceforth life will have for us a new meaning. It will be fraught with new opportunities and corresponding responsibilities.

> New occasions teach new duties,
>   Time makes ancient good uncouth;
> They must upward still and onward
>   Who would keep abreast of truth.
> Lo! before us gleam her campfires,
>   We ourselves must pilgrims be;
> Launch out, Mayflower!
>   And steer boldly through desperate winter sea.

